# COVID-19 Disease and Associated Thrombocytopenia: Pathogenesis and a Clue to the Etiology

**DOI:** 10.3390/diagnostics12051038

**Published:** 2022-04-20

**Authors:** Adil Abozaid Eissa

**Affiliations:** Department of Pathology, College of Medicine, University of Duhok, Duhok 42001, Iraq; adilkhr77@uod.ac; Tel.: +964-750-457-6051

**Keywords:** SARS-CoV-2 virus, COVID-19 disease, thrombocytopenia, IL-1Ra

## Abstract

(1) Background: Hospital mortality in patients suffering from SARS-CoV-2 infection has been associated with thrombocytopenia. The present study was conducted to establish the correlation of thrombocytopenia and the severity of infection. The impact of IL-1Ra gene polymorphism on the incidence and severity of thrombocytopenia was also studied. (2) Methods: Various biochemical parameters measured in all the 1200 enrolled patients included full blood counts, renal and liver function tests, iron study, inflammatory markers, and coagulation assays. A further 70 patients each were selected from the severe thrombocytopenic and non-thrombocytopenic patient groups to study the IL-1Ra gene polymorphism by RCR. (3) Results: Out of 1200 patients, 436 (36.3%) had thrombocytopenia. Among these patients, 118 (27.1%), 75 (17.2%), and 42 (9.6%) had mild, moderate, severe, and very severe thrombocytopenia, respectively. Severe cases mostly resulted from peripheral consumption (73.5%), hemo-phagocytosis (15.4%), and bone marrow suppression (11.11%). A statistically significant correlation was found between the occurrence and severity of thrombocytopenia with perturbated levels of inflammatory markers and the presence of comorbidities. The IL-1Ra∗3 variant was found to be significantly associated with thrombocytopenia. The IL-1Ra∗2 variant was significantly seen among controls. (4) Conclusions: The present study revealed a significant correlation between thrombocytopenia and the severity of COVID-19 disease. Moreover, the IL-1Ra∗3 variant of IL-1Ra gene was associated with thrombocytopenia.

## 1. Introduction

Thrombocytopenia is one of the most common hematological abnormalities seen in patients suffering from COVID-19 disease (SARS-CoV-2 infection) [1]. It results from different pathological mechanisms that range from mild platelets activation to the rapid removal of platelets from the circulation due to an autoimmune mechanism. The other causes include disseminated coagulation and microangiopathy or excessive utilization of platelets in an extensive inflammatory process affecting mainly the lung. Thrombocytopenia could also be a result of hematopoietic suppression in the bone marrow secondary to immunological activation with cytokine and interleukin storm that results from monocyte and histiocyte activation. Moreover, it could be due to hemo-phagocytosis or adverse effect of antiviral and other drugs; and/or direct invasion of hemopoietic elements through CD13 and CD66a receptors as documented in other coronavirus infections [2,3,4].

Thrombocytopenia is considered to be among the most important risk factors and is associated with increased morbidity and mortality in severe cases of infection, particularly in elderly patients. It usually denotes a widespread inflammatory response and overwhelming infection [5]. The role of platelets in infection and inflammation is crucial as they have been shown to play an important direct role directly in pathogenic recognition, sequestration, and elimination. They also act indirectly through the recruitment of neutrophils to the site of pathogenic invasion, and through modulation of granulocytic cells behavior, improving their phagocytic skills to kill microbial agents and encouraging exceptional protective functions, such as the production of Neutrophil Extracellular Traps (NETs) [6].

The severity of thrombocytopenia varies among different patients. This is mainly related to the response of the body to the infectious agents that are governed by the genetic heterogeneity and polymorphism of different genes responsible for an immune reaction to various infectious agents, including SARS-CoV-2 virus infection [7]. T-cells’ immune response is responsible for the regulation of the immune system through the secretion of many cytokines which play an important role in platelets activation. T cells also modulate the immune response after encountering various microbial agents, and excessive cytokines are released due to the dysregulation of the cytokine network, which is an additionally recognized factor that is associated with thrombocytopenia pathogenesis [8].

Infection with SARS-CoV-2 virus has been associated with abnormal levels of certain cytokines and chemokines including IL-6, IL-10, TNF-α, macrophage colony-stimulating factor (M-CSF), granulocyte (G-CSF), granulocyte-macrophage (GM-CSF), interferon-gamma-induced protein (IP-10), monocyte chemoattractant protein-1 (MCP-1), macrophage inflammatory protein 1-α (MIP 1-α), and TNF-α [9,10,11]. Release of the above cytokines is essential for the immediate immune response to infectious agents including viruses, bacteria, and other microorganisms in order to mobilize the effector cells and molecules to control the infection [12].

Cytokines are regulatory proteins that function in inflammation and the defense mechanism of the body by triggering immediate, innate non-specific antimicrobial effectors such as NK cells and macrophages. Functionally, cytokines are classified into two main categories: pro-inflammatory, such as IL-1 and TNF, and anti-inflammatory, such as IL-lRa and IL-10 molecules, while IL-6 has both of these properties [13]. Following microbial infection, the proinflammatory cytokines are induced rapidly, followed by the production of the anti-inflammatory cytokines. The genes responsible for cytokine production are polymorphic, and perturbation in the level of cytokines in inflammation, either via a prolonged strong or via a reduced response as secondary to gene expression, is deleterious, responsible for the antagonistic properties of the infectious and autoimmune diseases, and needs to be tightly regulated [7].

Studies have reported that both genetic and environmental factors have been involved in the pathogenesis of thrombocytopenia [7,14]. This has been specially attributed to interaction between epigenetic and genetic changes. Among various genetic factors, the polymorphism of cytokine genes has also been associated with thrombocytopenia [7]. Studies have reported that there is a strong association with genetic polymorphism in the cytokine genes, which effects secretion/production of cytokines and various diseased conditions such as infectious diseases, autoimmune diseases, and malignancy, both during the initiation of the disease and the disease course, along with response to the given treatment [15]. Genetic polymorphism of cytokine genes has been previously associated with the etiopathology of thrombocytopenia. This could be because, as discussed in detail above, abnormal functioning of cytokines contributes significantly to the pathogenesis of thrombocytopenia.

One such cytokine is the IL-1Ra regulatory cytokine which has been associated with immune thrombocytopenia previously. It has also been reported that polymorphic variants of the gene of this cytokine are responsible for the different susceptibility of individuals with different variants of many inflammatory diseases, such as rheumatoid arthritis and systemic lupus erythematosus [16]. Keeping this in view, in the present study genetic polymorphism studies were performed for the IL-1Ra gene in two groups of individuals, one infected with SARS-CoV-2 and having severe thrombocytopenia, and another group of patients who did not have thrombocytopenia. This will help in elucidating the correlation between the genetic variant of IL-Ra and thrombocytopenia. Moreover, since the first objective of the study is to establish the correlation between severity of COVID-19 and thrombocytopenia, disease severity will be correlated with the variant of IL-1Ra present in the thrombocytopenia group of patients.

## 2. Materials and Methods

### 2.1. Research Groups

A total of 1200 patients with confirmed SARS-CoV-2 infection were enrolled for the current study. These patients visited the molecular laboratory in Duhok/Iraq for diagnosis, then were followed during their course of disease in the hospital until complete recovery or death. Written informed consent was taken from all the recruited patients. The study was approved by the ethical committee of the University of Duhok/College of Medicine/Duhok/Iraq and followed the declaration of Helsinki for human studies. The complete disease history of all the patients recruited for the study was recorded and blood samples were collected to evaluate the complete blood count and blood morphology. The remaining blood was frozen to conduct later DNA studies. Blood samples were also collected for biochemical tests including renal functions tests, liver function tests, iron study, IL-6, C-Reactive Protein (CRP), Procalcitonin (PCT), Serum Amyloid A (SAA), and for coagulation assays including D-Dimer, prothrombin time, partial thromboplastin time and fibrinogen. All patients were followed during their stay in the hospital (whenever they required hospitalization) throughout the course of their diseases. Other investigations included CXR (PA view), CT scan, and physical examination.

Of all the enrolled patients, 70 patients with severe thrombocytopenia (platelets < 50 × 10^9^/L) and 70 without thrombocytopenia (platelets > 150 × 10^9^/L) were enrolled to study the impact of IL-1Ra gene polymorphism on the incidence and severity of thrombocytopenia.

### 2.2. DNA Isolation

DNA was extracted using a modified salting-out extraction method, which is reported to yield high purity DNA [17,18]. The sequence of primers, master-mix preparation, amplification, and gel electrophoresis was performed as mentioned elsewhere [7,19].

### 2.3. Statistical Analysis

All the collected data were analyzed using the Statistical Package for the Social Sciences software (SPSS version 24). For continuous variables, Student’s *t*-test was used, and for categorical variables the chi-square test or Fisher’s exact test was used. For variables such as platelet counts before and after hospitalization, paired *t*-test was used for comparison. *p*-value < 0.05 was considered statistically significant.

## 3. Results

Age of the enrolled patients ranged between 18 to 67 years with a median age of 34.3 years, and female to male ratio (712/488) was 1.46:1.

Of 1200 patients enrolled, 436 patients (36.3%) had thrombocytopenia with varying degrees of severity. The median age of the patients with thrombocytopenia was found to be higher than the median age of all the enrolled patients (56 ± 0.59). This was because the majority of elderly patients tend to have thrombocytopenia. A higher number of females was found to have thrombocytopenia (289/712 (40.6%)) compared to males (147/488 (30.1%)). Thrombocytopenia was found to be significantly high in middle-aged patients with other comorbidities, including diabetes mellitus and lower respiratory tract diseases (*p*-value of 0.024 and 0.015, respectively). There was no significant increase in thrombocytopenia in patients with heart disease (*p* = 0.33) (Table 1).

As for severity, the majority of the patients had mild thrombocytopenia that resolved spontaneously, followed by moderate and severe thrombocytopenia (Table 2). Severe thrombocytopenia was significantly associated with advanced age of more than 50 years (*p* = 0.016), female gender (*p* = 0.024), low hemoglobin, low WBC count, and low lymphocytes count with a *p*-value of 0.015, 0.045, and 0.0246, respectively.

Bone marrow examination results from patients with severe and very severe thrombocytopenia showed that thrombocytopenia most commonly resulted from peripheral consumption, followed by hemo-phagocytosis and BM suppression. Peripheral consumption could be attributed to extensive consumption in the inflammatory milieu that is the most possible mechanism, and less commonly due to peripheral consumption via real autoimmune mechanisms.

The incidence of thrombocytopenia and severity of thrombocytopenia were also associated significantly with increased CRP, SAA, and D-Dimer with *p* values consistently at < 0.005 (Table 3). Using paired *t*-test, improvement in biological markers such as CRP, SAA, and oxygen saturation was associated with increased platelet count.

Patients who needed admission due to hypoxia were found to have higher incidences of thrombocytopenia and of a more severe degree than those who did not require admission or oxygen therapy (*p* = 0.0114).

Patients who received steroids had lower incidence and severity of thrombocytopenia in comparison to those who did not receive steroids (*p* = 0.0214). Of all severe cases, 17 patients received eltrombopag 50 mg/day. These patients showed significant increase in platelet count after a median of 8 days to >50 × 10^9^/L.

For 25 patients with very severe thrombocytopenia, thrombopoietin receptors’ agonist (eltrombopag 50 mg once daily) caused a significant increase in platelet counts, as evident from the paired *t*-test (*p* = 0.036). However, it did not lead to any improvement in the mortality rate (*p* = 0.502), as 12 patients with eltrombopag and 10 patients without eltrombopag passed away.

The data regarding the molecular aspect of the study, i.e., the study of the polymorphism of the Interleukin-1 receptor antagonist (IL-1Ra) gene, has been represented in Table 4. Patients with the IL-1Ra*2 variant, known to be associated with impaired cytokine drives due to hyperactivity of the IL-1Ra gene, were seen significantly seen among controls (*p* value: 0.02). The IL-1Ra*3 variant known to be associated with excess cytokine secretion following exposure to foreign antigens due to impaired function of IL-1Ra gene as a result of tri-nucleotide repeat mutation, was seen significantly among patients with severe thrombocytopenia (*p* value: 0.047).

## 4. Discussion

In addition to their role in hemostasis, platelets perform several other functions due to the diverse actions of their contents and the antigens and receptors present on their surface. Platelets have been documented to play an important role in inflammation, modulation of the innate and adaptive immune response through adhesion, autophagy activity, and granule secretion, and the healing process following any injury. They also have Toll-like receptors (TLRs) expression which has a role in anti-tumor activity [5,20].

Infection with the SARS-CoV-2 virus induces a state of hyper-inflammation/autoimmunity dysregulation that induces extensive inflammation in suspected individuals. This inflammation is usually associated with an obvious elevation in inflammatory markers, and impairment of vital organs including bone marrow, manifested as pancytopenia with anemia, leukopenia, and thrombocytopenia. Understanding the fact that thrombocytopenia is an associated factor with SARS-CoV-2 infection, the present study was designed with a cohort population visiting the hospital for treatment for COVID-19 in order to understand if there is a correlation between the severity of the disease and thrombocytopenia, along with the association of thrombocytopenia with various other inflammatory and bio-chemical markers. The degree of thrombocytopenia as observed in the current study was concordant with inflammation, as severe thrombocytopenia has been significantly associated with marked elevation in the inflammatory markers, including CRP and SAA, in previous studies [21]. Initiation of the steroid did not improve the platelets count initially; however, after improvement in the inflammatory markers, this count was increased and this highlights the role of inflammatory cytokines such as TNF-α, INF-γ, and others in suppression of the marrow function and induction of thrombocytopenia [22].

Prognosis tended to be more severe in females, the elderly, and those with comorbidities as they are liable to have more severe infection, more frequent hospitalization, and a higher death rate from the increasing occurrence of complications, and these factors reflect the lower preservation and healing capacity of vital organs in such patients [23]. However, a few studies have shown contrary data to our findings [24]. Liu et al. found that thrombocytopenia was more frequent in the male population at the time of admission of COVID-19 patients in a hospital at Wuhan. However, similar to our data, they also reported that thrombocytopenia was associated with older age.

Thrombocytopenia has been recognized as a surrogate marker for higher risk of mortality in patients with septicemia, which is further associated with profound immune response and platelets activation secondary to the release of platelets’ contents [25]. Such platelet activation is usually of minimal significance in viral infection; however, with secondary bacterial infection that results from disruption of epithelial surface and superadded infection, profound inflammation may be encountered. This inflammation could exaggerate the immune response that could be explained by a high IL-6 level and IL-1Ra*3 genotype in such individuals.

Eltrombopag had been used previously to support patients with HCV infection, cirrhosis, and thrombocytopenia with successful results, as such patients have liver function impairment with a reduced level of thrombopoietin [26]. It has also been shown to play a role in aplastic anemia through activation of the remaining megakaryocytes or their role in hemopoietic stem cell stimulation [27]. These two mechanisms are related to the rise in relatively deficient thrombopoietin and hemopoietic stem cells activation, in addition to their role in suppression of excess inflammation through the TRL4 pathway, and might have worked together in raising the platelets count in the enrolled patients [25].

Polymorphism in the cytokines’ genes, particularly IL-1Ra regulatory cytokines, have been reported to bring about various responses in infected individuals, and make individuals with certain variants more susceptible to some inflammatory diseases, including alopecia areata, ankylosing spondylitis, SLE, rheumatoid arthritis, etc. [16]. Keeping this in view, I studied genetic polymorphism in the IL-1Ra gene was studied for any correlation with thrombocytopenia in COVID-19 patients. A significant association was found between the IL-1Ra∗3 variant and thrombocytopenia, as most of the patients in the thrombocytopenia group had the IL-1Ra∗3 variant. The control population taken for this comparison which was did not have thrombocytopenia was found have the IL-1Ra∗3 variant. These results were in line with previously published data by Yadav et al., who showed that in the case of immune thrombocytopenic purpura (ITP) which is reported to be one of the most common childhood autoimmune diseases, the IL-1Ra∗3 variant was associated with the etiology and the course of the disease [8].

Overall, the study suggests that thrombocytopenia is associated with disease severity in COVID-19 patients and with the IL-1Ra∗3 variant. It could be concluded from these findings that there exists a relationship between disease severity, the IL-1Ra∗3 variant and thrombocytopenia, which could be used as a strategy for disease prognosis and to further decide treatment plans for better recovery.

## 5. Conclusions

Thrombocytopenia in patients with SARS-CoV-2 infection is multifactorial and correlates significantly with the severity of the disease. In the present study, evaluation was made of the correlation between observed thrombocytopenia in COVD-19 patients and the severity of the disease, if any. It is well established that inflammatory responses and cytokines secreted by the T-cells have a major role in the disease pathogenesis of COVID-19 and these released cytokines determine the severity of the disease, as well its control by the immune response. Herein, this study attempted to evaluate the genetic polymorphism of the IL-1Ra cytokine gene, and how the different variants of this cytokine gene are associated with disease severity. Our study suggested that there exists a significant correlation between the severity of the SARS-CoV-2 infection and thrombocytopenia. Moreover, thrombocytopenia was found to be significantly high in patients with various comorbidities. The genetic polymorphism study revealed that the IL-1Ra∗3 variant of IL-1Ra was significantly associated with thrombocytopenia and the IL-1Ra∗2 variant was significantly seen among controls. All the data, if examined cumulatively, suggest that the presence of the IL-1Ra gene is associated with thrombocytopenia, which is further associated with disease severity. Therefore, the presence of the IL-1Ra∗3 variant serves as a biomarker for severe cases of COVID-19 and needs to be further explored for various diagnostic purposes.

## Figures and Tables

**Table 1:** Comorbidities among enrolled patients.

Comorbidities	No. (%)	Association with Thrombocytopenia *p*-Value
Diabetes	18 (1.5%)	0.024
Heart disease	6 (0.5%)	0.33
Respiratory diseases	13 (1.1%)	0.015
Others	11 (0.9%)	NA

**Table 2:** Degrees of thrombocytopenia and the most likely underlying mechanisms.

Degree of Thrombocytopenia	No. (%)	Most Likely Underlying Mechanisms	
Mild	201 (46.1%)		
Moderate	118 (27.1%)		
Severe	75 (17.2%)	Peripheral consumption	86 (73.5%)
Very severe	42 (9.6%)	Hemo-phagocytosis	18 (15.4%)
		B.M suppression	13 (11.1%)

**Table 3:** Biological markers among different groups of patients.

		Reference Ranges for the Test Used	Normal Platelets	Mild TCP	Moderate TCP	Severe TCP	Very Severe TCP	Normal vs. Severe *p* Value	Normal vs. Very Severe *p* Value
Platelet count (×10^9^/L)		150–450	>150	100–150	50–100	20–50	<20		
Hb. g/dL	Males	13–17	14.9 ± 1.16	14.8 ± 0.54	13.8 ± 2.13	11.9 ± 0.87	8.7 ± 1.1	0.046	<0.005
	Females	12–15	11.5 ± 0.69	11.6 ± 1.06	11.1 ± 1.12	9.7 ± 0.56	8.5 ± 0.98	0.036	<0.005
WBC count (×10^9^/L)		4.0–10.0	6.7 ± 0.23	4.9 ± 0.26	4.6 ± 0.48	2.9 ± 0.19	2.8 ± 0.88	0.006	<0.005
Lym. Count (×10^9^/L)		1–3	2.1 ± 0.10	1.8 ± 0.12	1.7 ± 0.08	0.4 ± 0.09	0.2 ± 0.06	0.005	0.026
IL-6 (pg/mL)		<7.0	5.2 ± 0.23	5.1 ± 0.18	10.3 ± 1.02	12.5 ± 1.11	19.2 ± 1.59	<0.005	<0.005
D. Dimer (ng/mL)		<500	456.2 ± 15.9	512.6 ± 15.7	725.6 ± 88.1	1216.2 ± 98.5	3256.5 ± 159.2	<0.005	<0.005
CRP (mg/L)		<10.0	10.2 ± 2.62	8.6 ± 3.65	12.6 ± 4.12	64.6 ± 14.74	256.2 ± 16.9	<0.005	<0.005
SAA (mg/L)		<10.0	2.7 ± 0.69	3.10 ± 4.9	12.6 ± 3.61	89.5 ± 10.25	317.3 ± 26.9	<0.005	<0.005
PCT µg/L		<0.05	0.2 ± 0.05	0.23 ± 0.58	0.85 ± 0.11	1.12 ± 1.02	5.12 ± 1.02	<0.005	<0.005
Creatinine (mg/dL)		<1.4	0.89 ± 0.06	0.79 ± 0.15	1.02 ± 0.06	1.18 ± 0.51	1.89 ± 1.0	0.256	0.119
GPT (U/L)		<31.0	59.2 ± 5.21	57.6 ± 5.9	64.1 ± 6.17	65.1 ± 4.16	84.5 ± 9.87	0.096	0.068
Albumin (g/dL)		3.5–5.5	4.96 ± 0.19	4.59 ± 0.24	4.17 ± 0.32	3.32 ± 0.20	3.16 ± 0.56	0.840	0.560
PT (seconds)		<13.0	13.5 ± 0.11	13.8 ± 0.94	15.2 ± 0.12	19.2 ± 0.19	19.5 ± 2.69	0.169	0.069
PTT (seconds)		26–40	26.5 ± 0.21	26.4 ± 1.02	27.1 ± 0.21	28.0 ± 0.56	36.4 ± 4.51	0.126	0.052
Fibrinogen (g/dL)		200–400	345.6 ± 19.54	352.2 ± 10.9	362.1 ± 12.5	456.2 ± 18.95	512.2 ± 32.65	0.146	0.099

**Table 4:** Frequency of different IL-1Ra variants among patients with severe thrombocytopenia and control group.

IL-1Ra	Variants	Cases	Controls	Total
	1,1	44 (62.9%)	46 (65.7%)	90 (64.3%)
	1,2	2 (2.9%)	6 (8.6%)	8 (5.7%)
	1,3	9 (12.9%)	4 (5.7%)	13 (9.3%)
	1,4	3 (4.3%)	1 (1.4%)	4 (2.9%)
	2,2	1 (1.4%)	3 (4.3%)	6 (4.3%)
	2,3	1 (1.4%)	1 (1.4%)	2 (1.4%)
	2,4	2 (2.9%)	6 (8.6%)	8 (5.7%)
	3,3	3 (4.3%)	1 (1.4%)	2 (1.4%)
	3,4	2 (2.9%)	1 (1.4%)	3 (2.1%)
	4,4	3 (4.3%)	1 (1.4%)	4 (2.9%)

## Data Availability

Data available on request.

## Abbreviations

PCR: Polymerase chain reaction, IL-6: Interleukin–6, CRP: C-Reactive Protein, PCT: Procalcitonin, SAA: Serum Amyloid A, IL-1Ra: Interleukin 1 receptor antagonist, PCT: Procalcitonin, Hb.: Hemoglobin, WBC: white blood cells, GPT: Glutamate pyruvate transaminase, PT: prothrombin Time, PTT: Partial thromboplastin time.
